# Beneficial Effects of Acetyl-DL-Leucine (ADLL) in a Mouse Model of Sandhoff Disease

**DOI:** 10.3390/jcm9041050

**Published:** 2020-04-08

**Authors:** Ecem Kaya, David A. Smith, Claire Smith, Barry Boland, Michael Strupp, Frances M. Platt

**Affiliations:** 1 Department of Pharmacology, University of Oxford, Mansfield Road, Oxford OX1 3QT, UKDave.smith@pharm.ox.ac.uk (D.A.S.); claire.fletcher@pharm.ox.ac.uk (C.S.); 2 Department of Pharmacology and Therapeutics, Western Gateway Building, College of Medicine and Health, University College Cork, T12XF62 Cork, Ireland; barry.boland@ucc.ie; 3 Department of Neurology and German Center for Vertigo and Balance Disorders, Ludwig Maximilians University of Munich, D-81377 Munich, Germany; Michael.Strupp@med.uni-muenchen.de

**Keywords:** Sandhoff disease, acetyl-DL-Leucine, Tanganil, substrate reduction therapy, metabolism

## Abstract

Sandhoff disease is a rare neurodegenerative lysosomal storage disease associated with the storage of GM2 ganglioside in late endosomes/lysosomes. Here, we explored the efficacy of acetyl-DL-leucine (ADLL), which has been shown to improve ataxia in observational studies in patients with Niemann–Pick Type C1 and other cerebellar ataxias. We treated a mouse model of Sandhoff disease (*Hexb^-/-^*) (0.1 g/kg/day) from 3 weeks of age with this orally available drug. ADLL produced a modest but significant increase in life span, accompanied by improved motor function and reduced glycosphingolipid (GSL) storage in the forebrain and cerebellum, in particular GA2. ADLL was also found to normalize altered glucose and glutamate metabolism, as well as increasing autophagy and the reactive oxygen species (ROS) scavenger, superoxide dismutase (SOD1). Our findings provide new insights into metabolic abnormalities in Sandhoff disease, which could be targeted with new therapeutic approaches, including ADLL.

## 1. Introduction

The GM2 gangliosidoses [1] are lysosomal storage diseases that include Tay–Sachs disease, Sandhoff disease and GM2 activator deficiency. Sandhoff disease is caused by mutations in *HEXB*, a gene that encodes the beta subunit of β-hexosaminidase, leading to storage of GM2 ganglioside and other glycoconjugate substrates in the central nervous system (CNS), resulting in progressive neurodegeneration, CNS inflammation and premature death [2]. Symptom onset can occur in infancy or during childhood/adolescence or adulthood [3]. Early-onset disease results from more disabling mutations that lead to attenuated residual enzyme activity and rapid disease progression, while later-onset forms with some residual enzyme activity progress more slowly [3]. Currently, there are no approved disease-specific treatments for Sandhoff disease. However, several experimental approaches have been evaluated over the years, including bone marrow transplantation, substrate reduction therapy (SRT) and gene therapy [4,5,6,7]. For example, the SRT drug, miglustat, reduced levels of GM2 and GA2 in a mouse model of Sandhoff disease (*Hexb*^-/-^), resulting in functional benefit [4,8]. In observational clinical studies, miglustat also provided some benefit to juvenile onset Sandhoff patients [9,10]. 

Acetyl-*DL*-leucine (ADLL) is a derivative of the branched chain amino acid leucine, which has been used since 1957 as a treatment for acute vertigo and vertiginous symptoms (Tanganil^®^). It is orally available and has an excellent safety profile; however, its mechanism(s) of action is not fully understood. Drug distribution of intravenous (i.v.) ADLL is rapid and prominent in brain regions responsible for balance, coordination and central vestibular compensation [11]. Electrophysiological studies on vestibular-related networks in a guinea pig model of vertigo suggested ADLL’s anti-vertigo effect might be due to its normalisation of abnormal neuronal membrane potentials [12]. ADLL has also been trialled in observational studies via oral administration in monogenic cerebellar ataxia patients, producing significant improvement in gait and other symptoms [13,14]. Another case study that adopted an injectable administration of ADLL for cerebellar ataxia did not find benefit [15], implying that pharmacokinetic parameters are important for its efficacy. Later, oral ADLL was trialled in patients with another lysosomal lipid storage disorder, Niemann–Pick Type C1 (NPC1), where ataxia and dysmetria were reported to improve [14,16]. In addition, cognitive improvements were anecdotally reported by parents of some NPC1 patients who received ADLL treatment, suggesting potential functional benefit beyond the cerebellar system [16]. ADLL was also found to correct lysosomal pathology in fibroblasts of Tangier disease patients [17]. Tangier disease is due to mutations in the ATP-binding cassette transporter subfamily A number 1 (*ABCA1*), and has convergent cellular pathology with NPC [17], suggesting that this modified leucine amino acid can display benefit as a disease modifier in diseases with different aetiologies. Recently, acetyl-L-leucine has been identified as the pharmacologically active enantiomer [18,19]. Currently, there are three ongoing trials with acetyl-L-leucine in NPC1, GM2 gangliosidosis and ataxia telangiectasia (clinicaltrials.gov NCT03759639, NCT03759665, NCT03759678, and EudraCT (2018-004331-71; 018-004406-25; 2018-004407-39)).

In this study, we explored the effect of ADLL (a 50:50 racemic mixture of acetyl-L-leucine (ALL) and acetyl-D-leucine (ADL) in a mouse model of Sandhoff disease (*Hexb*^-/-^). Effects of the stereoisomers individual enantiomers ALL and ADL were not investigated separately. When ADLL treatment was initiated at weaning (3 weeks of age) in *Hexb*^-/-^ mice, there was a modest but significant extension in life span, delayed onset of motor function impairment, and reduced lipid storage in the brain. We also explored ADLL’s potential mechanism of action, and its impact when used in combination with miglustat. In the *Hexb*^-/-^ mouse cerebellum, ADLL treatment increased autophagy and normalized key enzymes in the metabolism of glucose and glutamate. ADLL also showed an additive effect when combined with the disease-modifying drug, miglustat. These findings suggest that ADLL may be of benefit in diseases involving metabolic imbalance in the CNS.

## 2. Experimental Section

### 2.1. Mice

Sandhoff disease mice [20] (*Hexb*^-/-^) were bred as heterozygotes (*Hexb*^+/-^) to generate affected mice (*Hexb*^-/-^*)* and wild-type controls (*Hexb^+/+^)* and were housed under non-sterile conditions, with food and water available ad libitum. All experiments were conducted using protocols approved by the UK Home Office Animal Scientific Procedures Act, 1986. All animal works were conducted under the UK Home Office licensing authority, and the project license number is P8088558D. The ethical review committee with oversight of the research that was conducted using animals (mice) is the Committee on Animal Care and Ethical Review (ACER). No human subjects were involved in this study.

### 2.2. Drug Treatments

ADLL (Molekula #73891210) (0.1 g/kg/day) and miglustat (600 mg/kg/day) (Oxford GlycoSciences) were administered by mixing the drug in powdered chow (PicoLab Rodent Diet 20/5053, LabDiet). Mice were randomly assigned to treatment groups and staff were blinded to treatment when performing behavioural analysis. 

### 2.3. Behavioural Analysis

Weekly behavioural tests (9–14 weeks) were conducted in a blinded manner for the first cohort of mice. Gait analysis was performed using the CatWalk 10.5 system (Noldus Information Technology) according to the manufacturer’s instructions and five compliant runs were recorded per animal at each time point. The camera was set to 40 cm below the walkway, the walkway was approximately 4 cm wide, and detection settings were set to 14.05 camera gain and 0.12 green intensity. Motor function of mice was measured using a Ugo Basile RotaRod NG (Italy), starting from 1 to 10 rpm, and accelerating every 30 s by one rpm.

Bar crossing experiments were conducted to measure motor strength and coordination [21]. Briefly, a metal bar (1.2 mm in width and 26 cm in length) was suspended horizontally between two wooden supports, 30 cm in height, over a cushioned surface, and animals were allowed to grasp the centre of the bar with forepaws only, the tail was released, and the clock was started. Latency to cross or fall from the bar was scored, with a 180 s maximum to termination of each trial. A score was given for each animal between +180 and −180. If crossing time (CT) was greater than 0, then score = 180 − CT; if falling time (FT) was greater than 0, then score = FT − 180.

### 2.4. Tissue Handling

A second cohort of mice was sacrificed at 86 days of age (late symptomatic stage) and perfused with phosphate-buffered saline (PBS, pH 7.4) (Gibco #10010023). Tissues for biochemical analysis were snap frozen in ice-cold isopentane (−80 °C). Biochemical analyses were performed on water-homogenized tissues (mg/mL) and protein content was determined using a BCA protein assay (Thermo Fisher #23227) according to the manufacturer’s instructions.

### 2.5. Glycosphingolipid Measurements

Glycosphingolipids (GSLs) were measured according to published methods [22]. Briefly, GSLs were extracted from tissue homogenates in chloroform/methanol (C:M) (1:2 v/v) overnight at 4 °C. The mixture was centrifuged (3000 rpm/10 min) and 1 mL chloroform and 1 mL PBS were added to the supernatant and centrifuged (3000 rpm/10 min). The lower phase was dried under N_2_, resuspended in 50 uL C:M 1:3 v/v and combined with the upper phase. Subsequently, GSLs were recovered using ISOLUTE® C18 columns (Biotage, Sweden), pre-equilibrated with 4 × 1 mL MeOH and 2 × 1 mL H_2_O. Samples were washed with 3 × 1 mL H_2_O and eluted with 1 mL C:M 98:2, 2 × 1 mL C:M 1:3, and 1 mL MeOH. The column eluate was dried under N_2_, resuspended in 100 uL C:M 2:1, dried again under N_2_ and resuspended in ceramide glycanase (CGase) buffer (50 mM sodium acetate pH 5.5, 1 mg/mL sodium taurodeoxycholate). The 50 mU CGase was added, and samples were incubated at 37 °C overnight. Released oligosaccharides were labelled with anthranilic acid (2-AA) and purified by mixing with 1 mL acetonitrile:H_2_O (97:3 v/v), and added to Discovery DPA-6S columns (SUPELCO, # 52625-U) pre-equilibrated with 1 mL acetonitrile, 2 × 1 mL H_2_O, and 2 × 1 mL acetonitrile. Columns were washed with 2 × 1 mL acetonitrile:H_2_O (95:5 v/v) and eluted in 2 × 0.75 mL H_2_O. Samples were loaded 30:70 H_2_O:MeCN (v/v) for normal-phase (NP) HPLC. 2AA-labeled oligosaccharides were separated and quantified by NP-HPLC as previously described [22]. Separation was carried out at 30 °C using a Waters Alliance 2695 separation module, with excitation at 360 nm and emission at 425 nm, using a Waters 2475 fluorescence detector.

### 2.6. Western Blotting

Western blot analysis was carried out by homogenizing cerebellar hemispheres to 50 mg/mL (w/v) in RIPA buffer (Pierce RIPA Buffer, Thermo Fischer Scientific-89900) containing a protease/phosphatase inhibitor cocktail (Halt™ Protease and Phosphatase Inhibitor Cocktail-100X, Cat No: 78440), followed by a 30 min incubation on ice. Samples were centrifuged at 13,000 × *g* at 4 °C, and supernatants were retained for further BCA protein assay and Western blot. Protein extracts from cerebellar homogenates were obtained from samples lysed and stored in 1% Igepal CA, 0.5% sodium deoxycholate, 0.1% SDS, 1% protease-phosphatase inhibitor cocktail and 3X SDS blue loading buffer (New England Biolabs; #50-811-554). Samples were resolved using 4–12% SDS tris-glycine (Biorad) gel electrophoresis and transferred to a low-fluorescence PVDF membrane (Biorad Immun-Blot Low Fluorescence PVDF paper; #1620262) using a semidry transfer apparatus (Trans-Blot Turbo Transfer System (Biorad; #1704150)). The antibodies used are summarized in Table 1.

### 2.7. Image Acquisition and Quantification

Western blot image acquisition was conducted with the Odyssey Infrared Imaging system (Model No. 9120, LI-COR, Cambridge, UK). Fluorescence and luminescence quantifications were performed with Fiji Version 1.51g software (Image J) (http://fiji.sc/Fiji) (W. Rasband, NIH, USA).

### 2.8. Statistical ANALYSES

Differences between groups were identified either by one-way or two-way analyses of variances (ANOVA); comparisons of groups were made after two-way ANOVAs with Tukey’s multiple comparison tests. Statistical tests were performed using Prism 6 software (Graphpad v6, La Jolla, San Diego, CA, USA).

## 3. Results

### 3.1. The Effects of ADLL in Hexb^-/-^ Mice on Motor Function and Lipid Storage

The *Hexb^-/-^* mouse model of Sandhoff disease has a life span of approximately 16 weeks of age and is pre-symptomatic until approximately 6–8 weeks of age, and then progressively develops tremor and motor function deficits [8]. In the later stages of the disease (12–15 weeks), these mice become increasingly inactive and are unable to complete motor function tests, such as bar crossing [8]. They progressively accumulate GSLs, in particular GM2 and GA2, in the CNS and visceral organs, including the liver.

**Figure 1:**
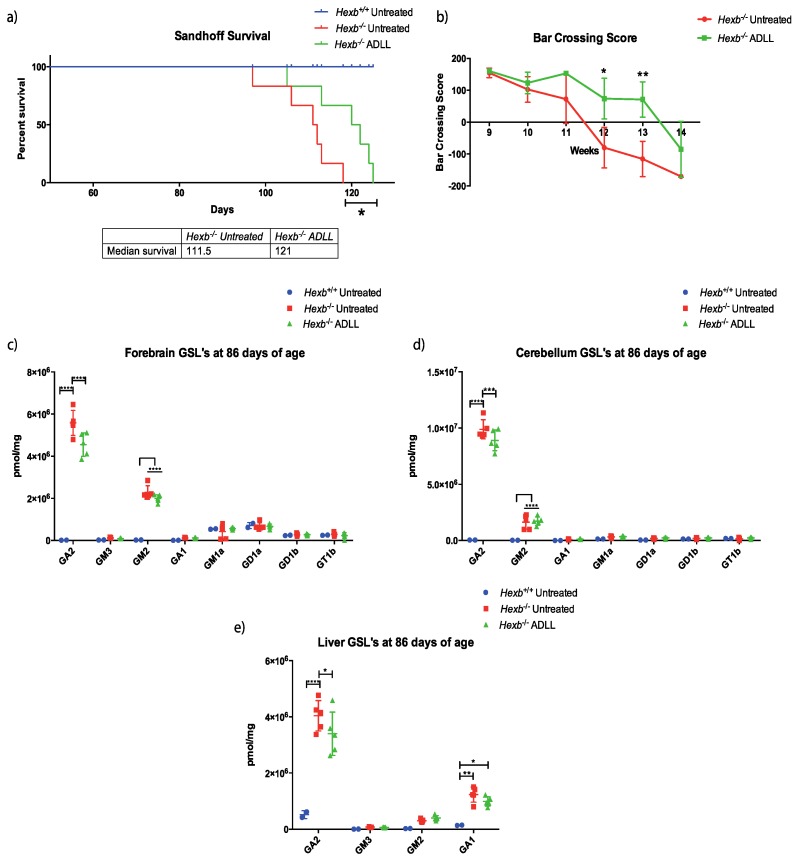
Effects of acetyl-DL-leucine on behavioural and biochemical parameters in Sandhoff disease (*Hexb^-/-^*) mice. The groups comprised Wild-type untreated (*Hexb^+/+^*), Sandhoff untreated (*Hexb^-/-^* UT), Sandhoff ADLL treated (*Hexb^-/-^*ADLL). In total, 2–5 animals for *Hexb^+/+^*, 5–6 animals for *Hexb^-/-^* UT and *Hexb^-/-^*ADLL. (**a**) Changes in life expectancy (%); *n* = 6 for *Hexb^-/--^*untreated and ADLL treated, *n* = 5 for *Hexb^+/+^*, mean ± SD, * *p* = 0.0247 (log-rank test). (**b**) Bar crossing score, *n* = 6, mean ± SD, * *p* = 0.0343, ** *p* = 0.0058 (two-way ANOVA). (**c**) GSL measurements of forebrain (brain) at 86 days of age. Mean ± SD, **** *p* < 0.0001, (two-way ANOVA). (**d**) GSL measurements of cerebellum at 86 days of age. Mean ± SD, *** *p* = 0.0002, ****p < 0.0001, (two-way ANOVA). (**e**) Liver GSLs at 86 days of age. Mean ± SD, * *p* < 0.0124, ** *p* = 0.0012, **** *p* < 0.0001, (two-way ANOVA).

*Hexb^-/-^* animals were either untreated or treated from weaning (3 weeks of age) with ADLL (0.1 mg/kg/day, equivalent to the dose used in observational clinical studies [13]). We observed a statistically significant extension in survival with ADLL (median increase of 8.5% (9.5 days), *p =* 0.0247) (Figure 1a). Bar-crossing performance (time to cross or fall) also improved significantly in the late-symptomatic stage of the disease (12 weeks: *p =* 0.0343; 13 weeks: *p* = 0.0058), which was indicative of slower disease progression (Figure 1b).

A second cohort of treated mice were sacrificed at 86 days of age (late-symptomatic stage) to measure changes in GSL levels in the forebrain, cerebellum and liver [8]. Although total GSL levels did not change with ADLL treatment in these organs (Supplementary Figure S1); some specific gangliosides were significantly reduced. For example, levels of GA2, the most accumulated ganglioside in the forebrain, were 18.5% lower in ADLL-treated *Hexb^-/-^* mice (*p* < 0.0001) relative to untreated *Hexb^-/-^* mice (Figure 1c). Similarly, GA2 levels were 10% lower in the cerebellum (*p* = 0.0002) (Figure 1d) and 15.8% lower in the liver in ADLL-treated *Hexb^-/-^* mice (*p* = 0.0121) (Figure 1e). Levels of other GSL species did not change significantly upon ADLL treatment in *Hexb^-/-^* mice (Figure 1c–e).

### 3.2. ADLL Increases Autophagy in Hexb^-/-^ Mice Cerebellum

**Figure 2:**
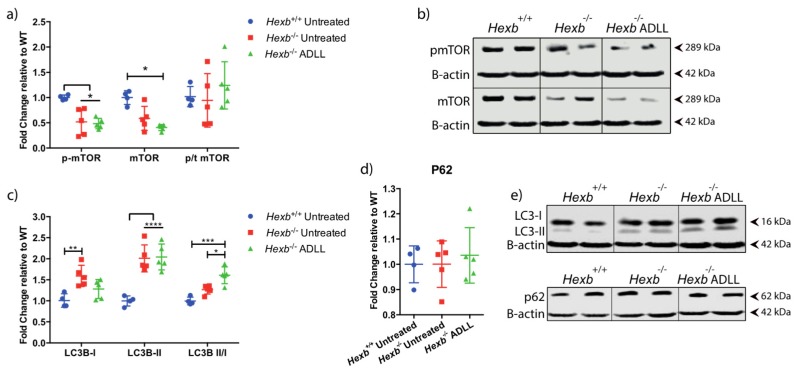
Effect of acetyl-*DL*-leucine on autophagy in the cerebellum of 12-week-old *Hexb^-/-^* mice. Wild-type untreated (*Hexb^+/+^*), Sandhoff untreated (*Hexb^-/-^* UT), ADLL (*Hexb^-/-^*ADLL). In total, four animals for *Hexb^+/+^*, and five animals for *Hexb^-/-^* UT and *Hexb^-/-^*ADLL. Proteins of interests were probed by Western blot and normalized to β actin. Fluorescence values were obtained with Fiji software and calculated relative to *Hexb^+/+^*. (**a**) mTOR, p-mTOR expressions and mTOR *p*:T rations, mean ± SD * *p* < 0.05, (two-way ANOVA). (**b**) Western blot images of *p*-mTOR and mTOR proteins and their β-actin loading controls. (**c**) LC3-I, LC3-II expressions, and ratio of LC3-II versus LC3-I, mean ± SD, *p = 0.0483, ** *p* = 0.0011, *** *p* = 0.0006, **** *p* < 0.0001 (two-way ANOVA). (**d**) p62 levels, mean ± SD (one-way ANOVA). (**e**) Western blot images of LC3s and p62 with their β-actin loading controls. Samples on the same blots were cut (inserted lines) for appropriate visual representation.

We conducted a series of experiments to explore the mechanism of action of ADLL in the CNS. Given that lipid reductions in the brain and the cerebellum showed similar trends (significant GA2 reduction) in response to ADLL, we focused on the cerebellum, as it is important for motor function, which is impaired in Sandhoff disease mice. To better understand how ADLL might mediate these disease-modifying effects in *Hexb^-/-^* mice, we first explored nutrient sensing and autophagy. The mechanistic target of rapamycin (mTOR) is a master regulator of anabolic metabolism and it suppresses catabolic pathways such as autophagy [23]. Leucine increases phosphorylation of mTOR and subsequent activation, as recently reported in NPC fibroblasts [24]. Phosphorylated mTOR (*p*-mTOR) levels were significantly decreased in *Hexb^-/-^* cerebellum (48.1%, *p* = 0.0456) compared to *Hexb^+/+^* cerebellum (Figure 2a,b). However, the ratio between phosphorylated and total non-phosphorylated enzyme (*p*:T) did not differ between *Hexb^+/+^* and *Hexb^-/-^* (Figure 2a,b). ADLL treatment reduced the protein levels of both *p*-mTOR and mTOR levels in the cerebellum. However, their levels, as well as the *p*-mTOR: total mTOR ratio did not reach statistical significance when compared to *Hexb^-/-^* untreated mice (Figure 2a,b). To determine the status of macroautophagy in the mouse brains, we measured changes in the autophagy marker, LC3, both in the cytosolic form (LC3-I) and autophagic vacuole (AV) bound form (LC3-II), along with the LC3 adaptor protein, p62. Both LC3-I and LC3-II were increased in *Hexb^-/-^*-untreated mice, compared to wild-type mice (LC3-I: 36.6%, *p =* 0.0011; LC3-II: 50.3%, *p <* 0.0001) (Figure 2c,e). However, the internal ratio of LC3-II:LC3-I (a more reliable indicator of autophagic events [25]) did not vary between *Hexb^+/+^* and *Hexb^-/-^* (Figure 2c,e). ADLL therapy resulted in a 19.5% reduction of LC3-I levels, but was not statistically significant (*p =* 0.0804), and no change in LC3-II levels compared to untreated *Hexb^-/-^* mice was observed (Figure 2c,e). The ratio of LC3-II:LC3-I increased significantly by 27.1% (*p =* 0.0483), suggesting an increased presence of AVs in ADLL-treated *Hexb^-/-^* cerebellum (Figure 2c,e). Expression of the LC3 adaptor protein, p62, did not differ between *Hexb^+/+^* and *Hexb^-/-^*-untreated cerebellums, and remained unaltered in ADLL-treated *Hexb^-/-^* mice (Figure 2d,e), suggesting that the ADLL-induced increase in AVs was not due to impaired clearance, but more likely an elevation of autophagic activity in *Hexb^-/-^* cerebellum.

### 3.3. ADLL Normalizes Metabolic Abnormalities in Sandhoff Mouse Cerebellum

#### 3.3.1. ADLL’s Effect on Branched Chain Amino Acid and Glutamate Metabolism

**Figure 3:**
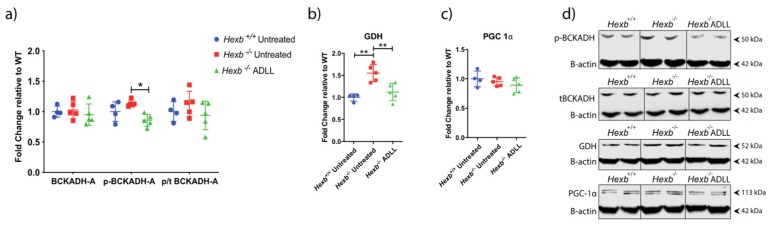
Effects of acetyl-DL-leucine on BCAA and glutamate and energy metabolism in the cerebellum of 12-week-old *Hexb^-/-^* mice. (**a**) BCKADH, *p*-BCKADH, *p*/t BCKADH expressions, mean ± SD, * *p* = 0.0494 (two-way ANOVA). (**b**) GDH expression. Mean ± SD ** *p* < 0.0057 (one-way ANOVA). (**c**) PGC1-α expression, mean ± SD (one-way ANOVA) (**d**) BCKADH, p-PCKADH, GDH and PGC1-α Western blot images with β-actin loading controls. Samples on the same blots were cut (inserted lines) for appropriate visual representation.

In addition to the known effects of the branched chain amino acid leucine on mTOR [26] and autophagy [27,28], leucine also plays a role in glutamine and glutamate utilisation when glucose metabolism is deficient/impaired [29,30]. Leucine catabolism provides a source of acetyl CoA [31]. Therefore, we investigated levels of branched chain keto acid dehydrogenase E1 alpha polypeptide (BCKDHA), which regulates the final step of leucine catabolism to acetyl CoA [32]. No difference in BCKDHA protein levels were found between *Hexb^+/+^* and *Hexb^-/-^* in the absence or presence of ADLL treatment (Figure 3a,d). There were also no statistical differences between healthy and diseased mice with regard to the inactive phosphorylated form of BCKDHA, or its *p*:T ratio (Figure 3a,d). Although ADLL decreased *p*-BCKADH levels significantly (24.3%, *p =* 0.0494), the absence of a change in its *p*:T ratio indicated that ADLL did not directly impact on BCKDHA activity (Figure 3a,d). Therefore, although there was a trend towards BCKADH activation with ADLL treatment, indicating enhanced ADLL catabolism, it failed to reach statistical significance.

Leucine also facilitates glutamate dehydrogenase (GDH)-mediated conversion of glutamate to α-ketoglutarate, which feeds into the TCA cycle [33]. GDH levels were increased in *Hexb^-/-^* mice compared to *Hexb^+/+^* mice (34%, *p =* 0.0014). However, ADLL supplementation decreased GDH expression by 26.6% in *Hexb^-/-^* cerebellum (*p =* 0.0056), restoring it to the normal range of *Hexb^+/+^* mice (Figure 3b,d). Whether this restoration is mediated directly by an effect on GDH, or indirectly through its reduction in lysosomal storage, remains unknown.

Leucine can affect mitochondria-mediated energy production through its effect on glucose utilisation [34]. Therefore, we tested the potential effect of ADLL treatment on energy metabolism by measuring the expression of the master regulator of mitochondrial biogenesis, peroxisome proliferator-activated receptor gamma coactivator 1-alpha (PGC1-α). No detectable differences in PGC1-α protein expression were observed in any group of mice evaluated (Figure 3c,d), which implied no alteration of mitochondrial biogenesis occurred with ADLL treatment.

#### 3.3.2. ADLL Promotes Anaerobic Glycolysis via PDK4 Up-Regulation

We then tested ADLL’s effect on glucose utilisation by examining enzymes that regulate aerobic glycolysis, via pyruvate dehydrogenase (PDH), and anaerobic glycolysis, via lactate dehydrogenase (LDH). PDH contains three subunits (E1, E2, E3), which convert the glycolysis product, pyruvate, to acetyl CoA, the prime substrate of the TCA cycle [35].

**Figure 4:**
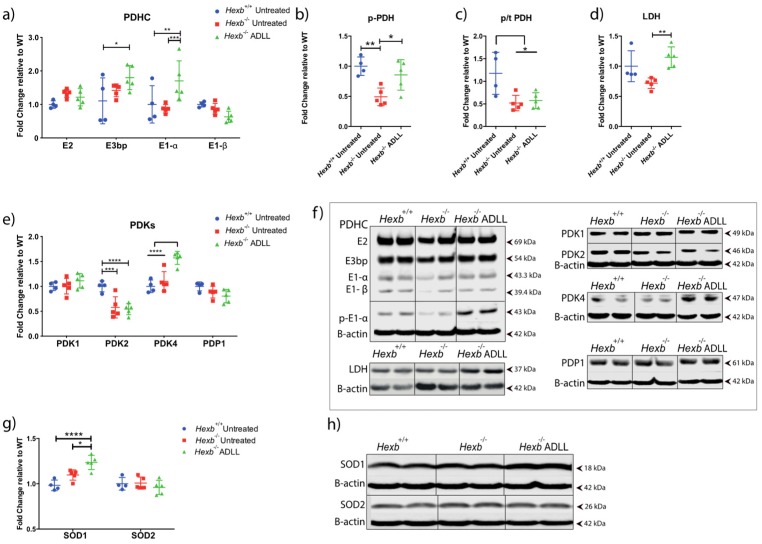
Effects of acetyl-DL-leucine on glucose and antioxidant metabolism in the cerebellum of 12-week-old *Hexb^-/-^* mice. (**a**) PDHC protein expression, mean ± SD, * *p* = 0.0105, ** *p* = 0.0097, *** *p* = 0.001 (two-way ANOVA). (**b**) *p*-PDH expression, mean ± SD, * *p* = 0.0310, ** *p* = 0.0062 (two-way ANOVA). (**c**) p-PDH/-tPDHE1α ratios, mean ± SD, * *p* < 0.023 (two-way ANOVA). (**d**) LDH expression, mean ± SD, ** *p* = 0.0071 (one-way ANOVA). (**e**) PDK 1-2-4 and PDP1 expression, mean ± SD, *** *p* = 0.0002, **** *p*< 0.0001 (two-way ANOVA). (**f**) Western blot images of PDHC, p-PDH, LDH, PDKs and PDP1 along with their β-actin controls. (**g**) SOD1 and SOD2 expression, mean ± SD, * *p* = 0.0216, ** *p* = 0.0121, **** *p* < 0.0001 (two-way ANOVA). (**h**) Western blot images of SOD1 and SOD2 and their β-actin loading controls. Samples on the same blots were cut (inserted lines) for appropriate visual representation.

By examining subunits of the pyruvate dehydrogenase complex (PDHC), we found no change in the expression of subunits E2, E3bp, E-α and E1-β in untreated *Hexb^-/-^* cerebellum compared to *Hexb^+/+^* mice (Figure 4a,f). However, E1-α subunit levels were increased by ADLL treatment in *Hexb^-/-^* mice compared to both *Hexb^+/+^* (41.1%, *p =* 0.0097) and *Hexb^-/-^* (48.8%, *p =* 0.0010) (Figure 4a,f). Considering PDH is negatively regulated by phosphorylation of its E1-α subunit [36], evidence of increased PDH activation in untreated *Hexb^-/-^* mice was indicated by significantly lower levels of *p*-E1-α (50.8%, *p* = 0.0062) (Figure 4b,f), compared to their *Hexb^+/+^* counterparts (55.9%, *p* = 0.0136) (Figure 4c,f).

ADLL treatment of *Hexb^-/-^* mice increased *p*-E1-α by 73.9% (*p* = 0.0310) relative to untreated *Hexb^-/-^* mice (Figure 4b,f), indicating a restoration in PDH activity. However, ADLL treatment did not change its proportionate activation (*p*:T ratio) in the *Hexb^-/-^* cerebellum (Figure 4c,f). Levels of LDH, the key enzyme in anaerobic glycolysis, did not differ between the *Hexb^+/+^* and *Hexb^-/-^* groups. However, ADLL significantly elevated LDH protein levels in the *Hexb^-/-^* cerebellum (59.6%, *p* = 0.0071) (Figure 4d,f). Thus, these results indicate that ADLL may have enhanced anaerobic glycolysis in *Hexb^-/-^* cerebellum.

The activity of the PDHC is regulated by 4 pyruvate dehydrogenase kinases (PDK 1,2,3,4), which phospho-inhibit the enzyme and 2 pyruvate dehydrogenase phosphatases (PDP1 and 2), which sustain its activation [37]. Considering ADLL significantly increased *p*-E1-α and LDH, we subsequently assessed PDH-modulating enzymes that are highly expressed in the cerebellum; namely, PDK1, 2, 4 and PDP1. Only PDK2 was significantly altered in the *Hexb^-/-^* cerebellum, being 42.2% lower than wild-type mice (*p* = 0.0002) (Figure 4e,f). ADLL treatment did not impact on PDK2 levels in *Hexb^-/-^* mice (Figure 4e,f). Nevertheless, PDK4 protein levels were significantly elevated in ADLL-treated *Hexb^-/-^* mice, compared to untreated *Hexb^-/-^* mice (30%, *p* < 0.0001) and *Hexb^+/+^* untreated (36.4%, *p* < 0.0001) (Figure 4e,f). PDP1 levels trended lower following ADLL treatment, but not significantly (Figure 4e,f). Considering ADLL induced an elevation of PDK4, which correlated with increased *p*-E1-α levels, these results indicate that ADLL’s mechanism of action may involve the normalisation of aerobic metabolism and facilitation of anaerobic pathways.

#### 3.3.3. ADLL Elevates Antioxidant SOD1 Levels

Leucine is known to reduce reactive oxygen species (ROS) via its enhancement of anaerobic glycolysis [38] and elevation of superoxide dismutase (SOD) levels [39]. We therefore explored ADLL’s impact on SOD1 and SOD2 in the cerebellum. SOD1 levels were similar in *Hexb^-/-^* and *Hexb^+/+^* mice. While ADLL treatment significantly increased SOD1 levels in the *Hexb^-/-^* cerebellum (12.59%, *p =* 0.0121) (Figure 4g,h), SOD2 levels were unchanged across all experimental groups (Figure 4g,h), these results indicate that ADLL treatment can induce antioxidant pathways.

### 3.4. Combination Treatment of Miglustat and ADLL Results in an Additive Extension of Lifespan in Hexb^-/-^ Mice

**Figure 5:**
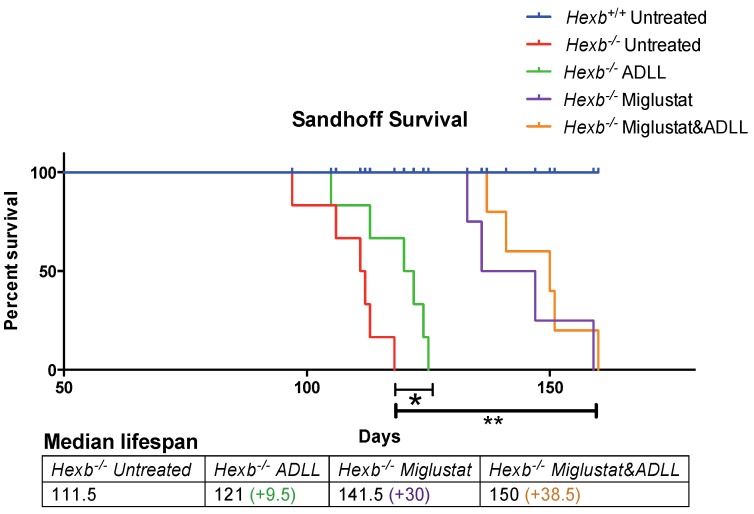
Evaluation of lifespan in miglustat alone or miglustat plus ADLL combination therapy in *Hexb^-/-^* mice. Wild-type (*Hexb^+/+^*) untreated (blue line), *n* = 5; Sandhoff (*Hexb^-/-^*) untreated (red line), *n* = 6; Sandhoff (*Hexb^-/-^*) ADLL treated (green line), *n* = 6; Sandhoff (*Hexb^-/-^*) miglustat treated (purple line), *n* = 4; Sandhoff (*Hexb^-/-^)* miglustat and ADLL treated (orange line), *n* = 5; changes in life expectancy (%). Mean ± SD, * *p* = 0.0247, ** *p <* 0.0039 (log-rank test). Median survivals are indicated in the table.

Finally, we combined ADLL with the substrate reduction therapy drug, miglustat (600 mg/kg/day), initiating both treatments from weaning (3 weeks of age). In agreement with previous studies [8], *Hexb^-/-^* mice had a median survival of 111.5 days, while miglustat therapy significantly increased the life expectancy by a median of 30 days (median survival 141.5 days, *p* = 0.0088) (Figure 5). ADLL monotherapy extended lifespan by a median of 9.5 days. However, miglustat and ADLL combination therapy resulted in 150 days survival time, i.e., 38.5 days longer than the untreated group (in median, *p* = 0.0014) (Figure 5), suggesting an additive effect of ADLL on miglustat therapy.

## 4. Discussion

L-Leucine not only serves as a building block for protein synthesis but is also a potent activator of the mammalian target of rapamycin (mTOR), involved in many cellular processes, including protein synthesis, nutrient sensing, cell growth, metabolism, and glucose, lipid and glutamate utilisation [26,34,38]. In this study, we explored the modified leucine amino acid, ADLL, for its potential therapeutic effects in a mouse model of Sandhoff disease, as it had previously provided beneficial effects in another lysosomal storage disease, NPC1 (observational clinical study) [16] and in Tangier disease patient cells [17]. Here, we found that ADLL modestly but significantly extended lifespan and significantly slowed disease progression in *Hexb^-/-^* mice. Furthermore, ADLL treatment reduced the most prominently accumulated glycosphingolipid, GA2, in both mouse liver and brain. In addition, ADLL increased autophagy, restored aerobic (PDH-dependent) and enhanced anaerobic (LDH-dependent) glycolysis, and returned the glutamate-metabolizing enzyme, GDH, to levels observed in *Hexb^+/+^* mice.

GA2 is the most abundant stored GSL in the *Hexb^-/-^* model (Figure 1). Only this lipid showed a statistically significant reduction in response to ADLL treatment. As other GSLs (e.g., GM2) were not reduced, these data suggest that ADLL is not acting as a substrate reduction therapy drug and may indicate instead that the GA2 reducing effect of ADLL occurs through an indirect mechanism. The precise mechanism will require further studies to elucidate.

Although 12-week-old Sandhoff cerebellum had no difference in their *p*:T mTOR ratio, ADLL treatment caused a slight reduction in total mTOR levels, and significantly elevated autophagy in *Hexb^-/-^* cerebellum. These findings may explain how ADLL induced the clearance of stored lipids through autophagy induction [40]. It is possible that the N-acetylation of leucine in ADLL blocks the interaction of leucine with the intracellular amino acid sensor, Sestrin 2, causing reduced mTOR activation, as was reported in HeLa cells [41,42], which could enhance autophagy.

Evidence that ADLL did not affect leucine catabolism was seen from the lack of alteration in BCKADH activity (*p*:T) in *Hexb^-/-^* cerebellum. However, an elevated level of glutamate dehydrogenase (GDH) is in agreement with a recent metabolomics study of *Hexb^-/-^* mice [43].

Mitochondrial energy metabolism is a major source of cellular ROS production [44], which reacts with lipids, proteins and DNA, and contributes to cell death [44]. Elevated oxidative stress as a result of ROS is a crucial pathology that contributes to neurodegeneration in Alzheimer’s disease, Parkinson’s disease and many other neurodegenerative conditions [45]. Similarly, Sandhoff disease has been found to manifest oxidative damage upon inflammation, which fosters neural death [46]. Leucine has been proposed to have differential effects on metabolism, depending on the catabolic and anabolic states. Several studies have observed that leucine-treated cells exhibited improvement in mitochondrial function and oxygen consumption [30]. However, another study found that leucine enhanced anaerobic glycolysis and therefore reduced OXPHOS-dependent ROS burden [38]. Similarly, leucine-treated rats on a high-fat diet (HFD) exhibited improved insulin sensitivity, reduced gluconeogenesis, improved lipid oxidation and mitochondrial function in obese/diabetic stage [47]. However, ADLL was found to contribute to mitochondrial dysfunction in high-fat diet (HFD)-fed rats at the early stage of insulin resistance [34,47], suggesting context-dependent effects.

**Scheme 1:**
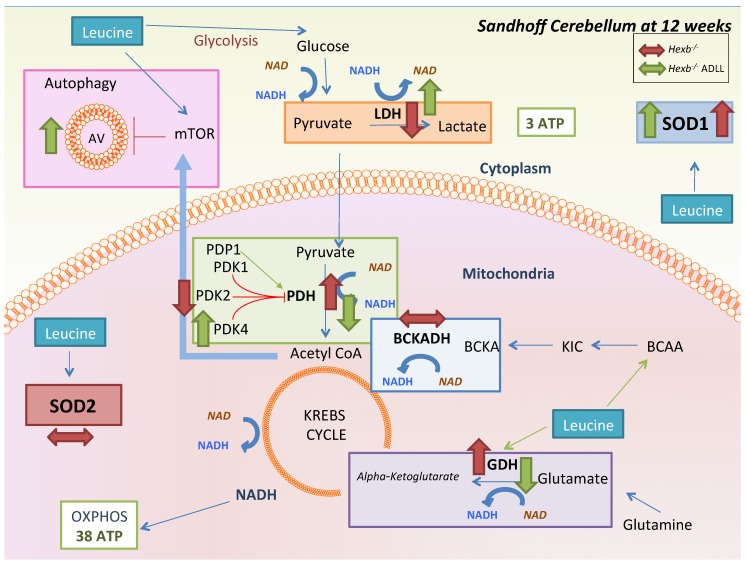
Summary of ADLL treatment effects on the *Hexb^-/-^* mouse cerebellum at 12 weeks of age. Investigated pathways that leucine can affect are displayed. Red arrows indicate the alterations in untreated *Hexb^-/-^* mice, while green arrows indicate the effect of pre-symptomatic ADLL treatment.

Recent metabolomic studies of Sandhoff disease mice and patient samples reported alterations in energy metabolism, where high levels of ROS and markers of oxidative stress were interpreted as increased respiratory chain activity [43]. In accordance with this, we found that PDH, a key indicator of aerobic respiration [48], was more active (55.9% reduction of *p*:T PDHE1-α levels) in *Hexb^-/-^* compared to *Hexb^+/+^* mice. This finding was consistent with a downregulation of PDK2, which phospho-inhibits PDH. ADLL treatment promoted deactivation of PDH by increasing PDK4 levels [49,50] and increasing LDH levels, thus normalizing glycolytic pathways to levels observed in *Hexb^+/+^* mice. Previous studies reported that PDK4 overexpression increased autophagy (elevated LC3-II levels) [51], and increased lactate production [36], which may explain the elevation in autophagy and LDH levels in *Hexb^-/-^* cerebellum with ADLL therapy. In a previous study, non-acetylated leucine had a similar effect in porcine intestinal epithelial cells, where it reduced the PDH-dependent OXPHOS pathway, and increased LDH-dependent glycolysis, thus reducing the OXPHOS-derived ROS burden [38]. A leucine-mediated reduction in ROS levels is consistent with the upregulation of the ROS scavenger, SOD1, which we observed with ADLL treatment. However, how ADLL upregulates PDK4, autophagy and lipid oxidation, and how this may link to a reduction of glycosphingolipid storage, remains unclear. Overall, changes in metabolic pathways with ADLL may be linked to leucine’s activatory effect on homeostatic AMP-activated protein kinase (AMPK), which regulates the change in nutrient utilisation, anabolic/catabolic shift and glycolysis preference [52]. AMPK can promote glycolysis as a survival mechanism upon oxidative stress [53]. AMPK can also promote oxidative metabolism via increasing mitochondrial biogenesis and OXPHOS capacity [54], correlating with a dual metabolic response upon leucine treatment in different systems. Furthermore, β-hydroxy-β-methyl butyrate, a minor metabolite of leucine, has been reported to stimulate AMPK activation insulin sensitivity and fat oxidation [55]. Therefore, the molecular mechanism of ADLL and its enantiomers, and their effects on AMPK merit further investigation in order to develop novel pharmacological approaches for lysosomal storage diseases. For a summary, see Scheme 1.

Miglustat reversibly inhibits glucosylceramide synthase, the enzyme that catalyses the first committed step in GSL biosynthesis, and was previously shown to slow disease progression in Sandhoff disease mice [8,9,10]. When ADLL and miglustat were combined, there was an additive effect in relation to extension in life expectancy, extending the life span by almost 5 weeks in total.

In summary, we have found that ADLL treatment of *Hexb^-/-^* mice partially reduced lipid storage, modestly improved behavioural parameters, significantly extended lifespan and provided additive benefit when combined with miglustat. This study provides mechanistic insights into novel pathological alterations in Sandhoff disease brain metabolism—some of which were corrected by ADLL treatment, highlighting the potential of this drug for treating Sandhoff disease and other lysosomal disorders.

## Figures and Tables

**Table 1:** List of antibodies and reagents used along with their usage conditions.

Protein/Epitope	Antibody Type	Host	Source	Catalogue Number	Dilution
β-Actin (HRP conjugated)	1^o^ Monoclonal	Mouse	Invitrogen	MA5-15739-HRP	1:15,000
BCKADH-A	1^o^ Polyclonal	Rabbit	abcam	ab90691	1:1000
p-BCKADH-A	1^o^ Polyclonal	Rabbit	abcam	ab200577	1:1000
GDH	1^o^ Monoclonal	Rabbit	Cell Signalling	D9F7P	1:1000
LC3B	1^o^ Polyclonal	Rabbit	abcam	ab51520	1:2000
LDHB	1^o^ Monoclonal	Mouse	abcam	ab85319	1:1500
mTOR	1^o^ Polyclonal	Rabbit	Cell Signalling	2972	1:500
*p*-mTOR (Ser 2448)	1^o^ Polyclonal	Rabbit	Cell Signalling	2971	1:500
P62	1^o^ Monoclonal	Mouse	abcam	ab56416	1:2000
*p*-PDH (Ser 293)	1^o^ Polyclonal	Rabbit	abcam	ab92696	1:500
PDH Complex (PDHC)	1^o^ Polyclonal	Mouse	abcam	ab110416	1:1000
PDK1	1^o^ Monoclonal	Mouse	abcam	ab110025	1:1000
PDK2	1^o^ Monoclonal	Rabbit	abcam	ab68164	1:1000
PDK4	1^o^ Monoclonal	Rabbit	abcam	ab214938	1:1000
PDP1	1^o^ Polyclonal	Rabbit	abcam	ab228578	1:1000
PGC-1 α	1^o^ Monoclonal	Mouse	Merck Millipore	ST1202	1:1500
SOD1	1^o^ Polyclonal	Rabbit	abcam	ab13498	1:1500
SOD2	1^o^ Polyclonal	Rabbit	abcam	ab13533	1:1500
Anti-Mouse IgG (H + L)	2^o^ Polyclonal	Goat	Licor-IRDye	925-32210	1:10,000
Anti-Rabbit IgG (H + L)	2^o^ Polyclonal	Goat	Licor-IRDye	925-68071	1:10000
Pierce ECL Substrate	-	-	Thermo Fisher	32106	-

## Supplementary Materials

The following are available online at https://www.mdpi.com/2077-0383/9/4/1050/s1, Figure S1: Total GSL levels at 12 weeks in *Hexb^-/-^* mice with and without treatment. Wild-type untreated (*Hexb^+/+^*), Sandhoff untreated (*Hexb^-/-^* UT), ADLL treated (*Hexb^-/-^*ADLL). *Hexb^+/+^(n =* 2), five animals for *Hexb^-/-^* UT and *Hexb^-/-^*ADLL, mean ± SD (two-way ANOVA).

## References

[1] Platt F.M., Boland B., van der Spoel A.C. (2012). The cell biology of disease: lysosomal storage disorders: the cellular impact of lysosomal dysfunction. J. Cell Biol..

[2] Wiederschain G.Y. (2002). The metabolic and molecular bases of inherited disease. Biochem..

[3] Maegawa G.H.B., Stockley T., Tropak M., Banwell B., Blaser S., Kok F., Giugliani R., Mahuran D., Clarke J.T.R. (2006). The Natural History of Juvenile or Subacute GM2 Gangliosidosis: 21 New Cases and Literature Review of 134 Previously Reported. Pediatrics.

[4] Butters T.D., Dwek R.A., Platt F.M. (2005). Imino sugar inhibitors for treating the lysosomal glycosphingolipidoses. Glycobiology.

[5] Wada R., Tifft C.J., Proia R.L. (2000). Microglial activation precedes acute neurodegeneration in Sandhoff disease and is suppressed by bone marrow transplantation. Proc. Natl. Acad. Sci. USA.

[6] Jeyakumar M., Norflus F., Tiff C.J., Cortina-Borja M., Butters T.D., Proia R.L., Perry V.H., Dwek R.A., Platt F.M. (2001). Enhanced survival in Sandhoff disease mice receiving a combination of substrate deprivation therapy and bone marrow transplantation. Blood.

[7] Begoacachon-González M., Wang S.Z., McNair R., Bradley J., Lunn D., Ziegler R., Cheng S.H., Cox T.M. (2012). Gene transfer corrects acute GM2 gangliosidosispotential therapeutic contribution of perivascular enzyme flow. Mol. Ther..

[8] Jeyakumar M., Butters T.D., Cortina-Borja M., Hunnam V., Proia R.L., Perry V.H., Dwek R.A., Platt F.M. (1999). Delayed symptom onset and increased life expectancy in Sandhoff disease mice treated with N-butyldeoxynojirimycin. Proc. Natl. Acad. Sci. USA.

[9] Tallaksen C.M.E., Berg J.E. (2009). Miglustat therapy in juvenile Sandhoff disease. J. Inherit. Metab. Dis..

[10] Wortmann S.B., Lefeber D.J., Dekomien G., Willemsen M.A.A.P., Wevers R.A., Morava E. (2009). Substrate deprivation therapy in juvenile Sandhoff disease. J. Inherit. Metab. Dis..

[11] Benard P., Cousse H., Bengonei T., Germaini C. (2001). Autoradiography in Brain of Macaca Iesciculeris Monkeys after injection of Acetyl-DL-Leucine [ 2 _ 14C ] ( Tanganil®). Eur. J. Drug Metab. Pharmacokinet..

[12] Vibert N., Vidal P.-P. (2001). *In vitro* effects of acetyl-DL-leucine (Tanganil®) on central vestibular neurons and vestibulo-ocular networks of the guinea-pig. Eur. J. Neurosci..

[13] Schniepp R., Strupp M., Wuehr M., Jahn K., Dieterich M., Brandt T., Feil K. (2016). Acetyl-DL-leucine improves gait variability in patients with cerebellar ataxia-a case series. Cerebellum & ataxias.

[14] Cortina-Borja M., Te Vruchte D., Mengel E., Amraoui Y., Imrie J., Jones S.A., I Dali C., Fineran P., Kirkegaard T., Runz H. (2018). Annual severity increment score as a tool for stratifying patients with Niemann-Pick disease type C and for recruitment to clinical trials. Orphanet J. Rare Dis..

[15] Pelz J.O., Fricke C., Saur D., Classen J. (2015). Failure to confirm benefit of acetyl-dl-leucine in degenerative cerebellar ataxia: a case series. J. Neurol..

[16] Bremova T., Malinova V., Amraoui Y., Mengel E., Reinke J., Kolnikova M., Strupp M. (2015). Acetyl-DL-leucine in Niemann-Pick type C: A case series. Neurology.

[17] Colaco A., Kaya E., Adriaenssens E., Davis L.C., Zampieri S., Fernández-Suárez M.E., Tan C.Y., Deegan P., Porter F.D., Galione A. (2019). Mechanistic convergence and shared therapeutic targets in Niemann-Pick disease type C and Tangier disease. J. Inherit. Metab. Dis..

[18] Te-Vruchte D., Galione A., Strupp M., Mann M. (2019). Effects of N-Acetyl-Leucine and its enantiomers in Niemann-Pick disease type C cells Danielle. bioRxiv Prepr..

[19] Churchill G.C., Strupp M., Galione A., Platt F.M. (2020). Unexpected differences in the pharmacokinetics of N-acetyl-DL-leucine enantiomers after oral dosing and their clinical relevance. PLoS One.

[20] Sango K., Yamanaka S., Hoffmann A., Okuda Y., Grinberg A., Westphal H., McDonald M.P., Crawley J.N., Sandhoff K., Suzuki K. (1995). Mouse models of Tay–Sachs and Sandhoff diseases differ in neurologic phenotype and ganglioside metabolism. Nat. Genet..

[21] Barclay L.L., Gibson G.E., Blass J.P. (1981). The string test: An early behavioral change in thiamine deficiency. Pharmacol. Biochem. Behav..

[22] Neville D.C.A., Coquard V., Priestman D.A., Te Vruchte D.J.M., Sillence D.J., Dwek R.A., Platt F.M., Butters T.D. (2004). Analysis of fluorescently labeled glycosphingolipid-derived oligosaccharides following ceramide glycanase digestion and anthranilic acid labeling. Anal. Biochem..

[23] Jung C.H., Ro S.H., Cao J., Otto N.M., Kim D.H. (2010). MTOR regulation of autophagy. FEBS Lett..

[24] Yanagisawa H., Ishii T., Endo K., Kawakami E., Nagao K., Miyashita T., Akiyama K., Watabe K., Komatsu M., Yamamoto D. (2017). L-leucine and SPNS1 coordinately ameliorate dysfunction of autophagy in mouse and human Niemann-Pick type C disease. Sci. Rep..

[25] Mizushima N., Yoshimori T. (2007). How to interpret LC3 immunoblotting. Autophagy.

[26] Zhang Y., Guo K., Leblanc R.E., Loh D., Schwartz G.J., Yu Y.H. (2007). Increasing Dietary Leucine Intake Reduces Diet-Induced Obesity and Improves Glucose and Cholesterol Metabolism in Mice via Multimechanisms. Diabetes.

[27] Klionsky D.J., Emr S.D. (2000). Autophagy as a regulated pathway of cellular degradation. Science.

[28] Yamamoto A., Cremona M.L., Rothman J.E. (2006). Autophagy-mediated clearance of huntingtin aggregates triggered by the insulin-signaling pathway. J. Cell Biol..

[29] Yudkoff M. (1997). Brain metabolism of branched-chain amino acids. Glia.

[30] Kennedy B.E., Hundert A.S., Goguen D., Weaver I.C.G., Karten B. (2016). Presymptomatic Alterations in Amino Acid Metabolism and DNA Methylation in the Cerebellum of a Murine Model of Niemann-Pick Type C Disease. Am. J. Pathol..

[31] Harris A., Paxton R., Powell S.M., Gillim S.E. (1985). Physiological covalent regulation of rat liver Branched-Chain a-Ketoacid Dehydrogenase. Arch. Biochem. Biophys..

[32] Harris R.A., Joshi M., Jeoung N.H., Obayashi M. (2005). Overview of the molecular and biochemical basis of branched-chain amino acid catabolism. J. Nutr..

[33] Erecińska M., Nelson D. (1990). Activation of Glutamate Dehydrogenase by Leucine and its nonmetabolizable analogue in rat brain synaptosomes. J. Neurochem..

[34] Pedroso J.A.B., Zampieri T.T., Donato J. (2015). Reviewing the effects of l-leucine supplementation in the regulation of food intake, energy balance, and glucose homeostasis. Nutrients.

[35] Park S., Jeon J.H., Min B.K., Ha C.M., Thoudam T., Park B.Y., Lee I.K. (2018). Role of the pyruvate dehydrogenase complex in metabolic remodeling: Differential pyruvate dehydrogenase complex functions in metabolism. Diabetes Metab. J..

[36] Zhang S., Hulver M.W., McMillan R.P., Cline M.A., Gilbert E.R. (2014). The pivotal role of pyruvate dehydrogenase kinases in metabolic flexibility. Nutr. Metab..

[37] Gray L.R., Tompkins S.C., Taylor E.B. (2014). Regulation of pyruvate metabolism and human disease. Cell. Mol. Life Sci..

[38] Hu J., Nie Y., Chen S., Xie C., Fan Q., Wang Z., Long B., Yan G., Zhong Q., Yan X. (2017). Leucine reduces reactive oxygen species levels via an energy metabolism switch by activation of the mTOR-HIF-1α pathway in porcine intestinal epithelial cells. Int. J. Biochem. Cell Biol..

[39] Cojocaru E., Filip N., Ungureanu C., Filip C., Danciu M. (2014). Effects of Valine and Leucine on Some Antioxidant Enzymes in Hypercholesterolemic Rats. Health (Irvine. Calif)..

[40] Lieberman A.P., Puertollano R., Raben N., Slaugenhaupt S., Walkley S.U., Ballabio A. (2012). Autophagy in lysosomal storage disorders. Autophagy.

[41] Nagamori S., Wiriyasermkul P., Okuda S., Kojima N., Hari Y., Kiyonaka S., Mori Y., Tominaga H., Ohgaki R., Kanai Y. (2016). Structure-activity relations of leucine derivatives reveal critical moieties for cellular uptake and activation of mTORC1-mediated signaling. Amino Acids.

[42] Wolfson R.L., Chantranupong L., Saxton R.A., Shen K., Scaria S.M., Cantor J.R., Sabatini D.M. (2016). Sestrin2 is a leucine sensor for the mTORC1 pathway. Science (80-. )..

[43] Ou L., Przybilla M.J., Whitley C.B. (2018). Metabolomics profiling reveals profound metabolic impairments in mice and patients with Sandhoff disease. Mol. Genet. Metab..

[44] Murphy M.P. (2009). How mitochondria produce reactive oxygen species. Biochem. J..

[45] Liu Z., Zhou T., Ziegler A.C., Dimitrion P., Zuo L. (2017). Oxidative Stress in Neurodegenerative Diseases: From Molecular Mechanisms to Clinical Applications. Oxid. Med. Cell. Longev..

[46] Jeyakumar M., Thomas R., Elliot-Smith E., Smith D.A., Van der Spoel A.C., D’Azzo A., Perry V.H., Butters T.D., Dwek R.A., Platt F.M. (2003). Central nervous system inflammation is a hallmark of pathogenesis in mouse models of GM1 and GM2 gangliosidosis. Brain.

[47] Liu R., Li H., Fan W., Jin Q., Chao T., Wu Y., Huang J., Hao L., Yang X. (2017). Leucine supplementation differently modulates branched-chain amino acid catabolism, mitochondrial function and metabolic profiles at the different stage of insulin resistance in rats on high-fat diet. Nutrients.

[48] Zheng X., Boyer L., Jin M., Mertens J., Kim Y., Ma L., Ma L., Hamm M., Gage F.H., Hunter T. (2016). Metabolic reprogramming during neuronal differentiation from aerobic glycolysis to neuronal oxidative phosphorylation. Elife.

[49] Vary T.C. (1996). Sepsis-induced alterations in pyruvate dehydrogenase complex activity in rat skeletal muscle: Effects on plasma lactate. Shock.

[50] Patel M.S., Korotchkina L.G. (2001). Regulation of mammalian pyruvate dehydrogenase complex by phosphorylation: complexity of multiple phosphorylation sites and kinases. Exp. Mol. Med..

[51] Mariño G., Pietrocola F., Eisenberg T., Kong Y., Malik S.A., Andryushkova A., Schroeder S., Pendl T., Harger A., Niso-Santano M. (2014). Regulation of autophagy by cytosolic Acetyl-Coenzyme A. Mol. Cell.

[52] Hardie D.G. (2011). Sensing of energy and nutrients by AMP-activated protein kinase. Am. J. Clin. Nutr..

[53] Wu S.B., Wei Y.H. (2012). AMPK-mediated increase of glycolysis as an adaptive response to oxidative stress in human cells: Implication of the cell survival in mitochondrial diseases. Biochim. Biophys. Acta - Mol. Basis Dis..

[54] Chaube B., Malvi P., Singh S.V., Mohammad N., Viollet B., Bhat M.K. (2015). AMPK maintains energy homeostasis and survival in cancer cells via regulating p38/PGC-1α-mediated mitochondrial biogenesis. Cell Death Discov..

[55] Bruckbauer A., Zemel M.B. (2013). Synergistic effects of metformin, resveratrol, and hydroxymethylbutyrate on insulin sensitivity. Diabetes, Metab. Syndr. Obes. Targets Ther..

