# Supplementary figures and images for: Beneficial Effects of Acetyl-DL-Leucine (ADLL) in a Mouse Model of Sandhoff Disease
Source: J Clin Med. 2020 Apr 8;9(4):1050. doi: 10.3390/jcm9041050 (PMC7230825; doi:10.3390/jcm9041050)

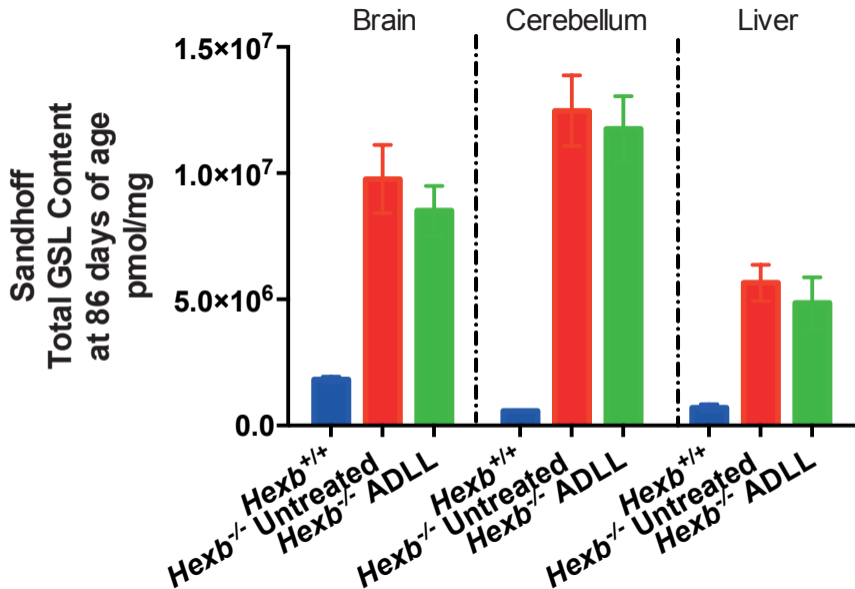

Supplement: Supplementary file 1 [file jcm-09-01050-s001.pdf]
